# Association between pemphigus and systemic lupus erythematosus: a systematic review and meta-analysis^☆^^☆☆^

**DOI:** 10.1016/j.abd.2020.07.009

**Published:** 2021-02-01

**Authors:** Vignesh Ramachandran, Kevin Phan, Saxon D. Smith

**Affiliations:** a Department of Dermatology, Baylor College of Medicine, Houston, Texas, United States; b Department of Dermatology, Liverpool Hospital, Sydney, Australia; c University of New, South Wales, Sydney, Australia; d Department of Dermatology, The Dermatology & Skin Cancer Centre, Sydney, Australia

*Dear Editor,*

Pemphigus defines a group of IgG-mediated autoimmune conditions targeting the squamous epithelium of skin and oral mucosa manifesting as intraepidermal blisters and erosions, which can be severely debilitating. The autoimmune etiology and chronicity of pemphigus disorders have spurred investigation into its association with other autoimmune conditions. Some studies note co-existence of pemphigus and systemic lupus erythematosus (SLE). Whether a true association exists or not is unknown. The authors present a systematic review and meta-analysis to assess the association between pemphigus and SLE.

Following PRISMA guidelines, searches were performed using PubMed, Cochrane Central Register of Controlled Trials, Cochrane Database of Systematic Reviews, Ovid MEDLINE, ACP Journal Club, and Database of Abstracts of Review of Effectiveness from their inception dates to August 2019. Search terms used were the following: “pemphigus foliaceus,” “pemphigus vulgaris,” or “pemphigus” in conjunction with “systemic lupus erythematosus,” “lupus,” or “SLE.” Studies included compared SLE cases in pemphigus patients *vs.* controls. Pemphigus cases included its subtypes (pemphigus vulgaris, pemphigus vegetans, pemphigus foliaceus, pemphigus erythematosus, and drug-induced pemphigus). Studies were excluded if clinical diagnosis was not determined by either direct or indirect immunofluorescence staining or enzyme-linked immunosorbent assay. Case reports, reviews, and studies without controls were also excluded. The odds ratio (OR) was calculated using a random effects model considering the baseline study heterogeneity as assessed with the I statistic. As a supplemental statistic, the number needed to treat (NNT) was calculated using the OR and the prevalence rate of SLE in North America (241/100,000 people, 0.241%) as a surrogate for the patient expected event rate (PEER).^1^ Review Manager v. 5.3 (Cochrane Collaboration – Oxford, United Kingdom) was the statistical software used.

There were 661 references identified; after exclusion of duplicate or irrelevant references, four studies were included (Table 1).^2^, ^3^, ^4^, ^5^ A statistically significant association between pemphigus and SLE was noted (OR = 2.16, 95% CI 1.09–4.25, p = 0.03), with heterogeneity (I^2^ = 50%) (Fig. 1). The NNT was calculated as 405 using North American SLE prevalence rates as a surrogate for PEER and the OR of 2.16.^1^

**Table 1:** Characteristics of studies that evaluated the association between pemphigus and systemic lupus erythematosus.

Author, Journal	Title	Location	Design	Number of cases	Number of controls
Chiu, Eur J Dermatol. 2017 Aug 1;27(4):375-381.^2^	Comorbid autoimmune diseases in patients with pemphigus: a nationwide case-control study in Taiwan	Taiwan	Case-control	1,998	7,992
Hsu, Br J Dermatol. 2016 Jun; 174(6):1290-8.^3^	Comorbidities and inpatient mortality for pemphigus in the United States.*	USA	Cross-sectional	6,406	87,033,305
Kridin, J Am Acad Dermatol. 2017 Dec; 77(6):1174-1175.^4^	Association between pemphigus and psoriasis: A population-based large-scale study.	Israel	Cross-sectional & meta-analysis	1,985	9,874
Parameswaran, Br J Dermatol. 2015 Mar; 172(3):729-38.^5^	Identification of a new disease cluster of pemphigus vulgaris with autoimmune thyroid disease, rheumatoid arthritis, and type I diabetes*	Global (primarily United States)	Cross-sectional & meta-analysis	230	General population statistics (Center for Disease Control and Prevention)

**Figure 1:**
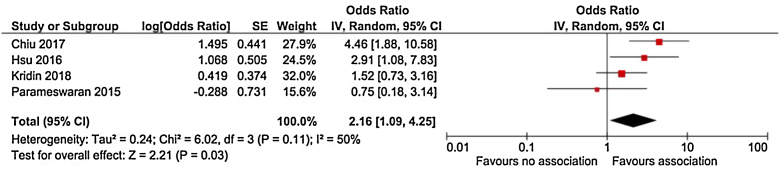
Forest plot representing the association between pemphigus and systemic lupus erythematosus (p = 0.03).

An autoimmune diathesis describes the association of autoimmune conditions with one another. Although literature describing co-existence of pemphigus with SLE are limited, pemphigus erythematosus (also known as Senear-Usher syndrome), an overlap syndrome between pemphigus vulgaris (PV) and SLE, was described in 1926.^6^ This may highlight an underlying pathophysiologic link between the conditions. Serologic studies demonstrate that one-third of PV cases display ANA-positivity with homogenous pattern, which is associated with active SLE.^7^ Furthermore, a recent genome-wide association study identified novel CD4+ T-cells pathways between pemphigus and SLE in addition to characterizing IRF8 and STAT1 as key regulatory genes.^8^ Both pemphigus and SLE display many antibodies. A proposed mechanism between such autoimmune conditions is epitope spreading, wherein the initial autoinflammatory response exposes new antigens, feeding into a subsequent autoimmune reaction.^9^

Limitations of this study include lack of data concerning immunopathological subtypes, clinical features, disease severity, chronology of diagnoses (*i.e.*, which diagnosis preceded the other), treatment-related information, and a true PEER value to obtain a more accurate NNT. Additionally, different study types, cohort sizes, and patient demographics may influence statistics.

In conclusion, an association was found between pemphigus and SLE, albeit an uncommon clinical occurrence. Nevertheless, clinicians caring for patients with pemphigus should be aware of this association and further research is required to elucidate the molecular basis of this association, more specifically specify its clinical significance and prevalence, and possibly identify optimal therapeutic strategies for patients with coexistence of both conditions.

## Financial support

None declared.

## Authors’ contributions

Vignesh Ramachandran: Approval of the final version of the manuscript; study conception and planning; preparation and writing of the manuscript; data collection, analysis, and interpretation; effective participation in research orientation; critical review of the literature; critical review of the manuscript.

Kevin Phan: Statistical analysis; approval of the final version of the manuscript; study conception and planning; data collection, analysis, and interpretation; effective participation in research orientation; critical review of the literature; critical review of the manuscript.

Saxon D. Smith: Approval of the final version of the manuscript; study conception and planning; preparation and writing of the manuscript; effective participation in research orientation; critical review of the manuscript.

## Conflicts of interest

None declared.
